# *In vivo* fluorescence imaging of conjunctival goblet cells

**DOI:** 10.1038/s41598-019-51893-4

**Published:** 2019-10-29

**Authors:** Seonghan Kim, Seunghun Lee, Hoonchul Chang, Moses Kim, Myoung Joon Kim, Ki Hean Kim

**Affiliations:** 1 Department of Mechanical Engineering, Pohang University of Science and Technology, 77 Cheongam-ro, Nam-gu, Pohang, Gyeoungbuk 37673 Republic of Korea; 2 Division of Integrative Biosciences and Biotechnology, Pohang University of Science and Technology, 77 Cheongam-ro, Nam-gu, Pohang, Gyeoungbuk 37673 Republic of Korea; 3 Department of Ophthalmology, Ulsan College of Medicine, Asan Medical Center, 88 Olympic-ro 43-gil, Songpa-gu, Seoul, 05505 Republic of Korea; 4 Renew Seoul Eye Center, 528 Teheran-ro, 4th Floor, Gangnam-gu, Seoul, 16181 Republic of Korea

**Keywords:** Translational research, Confocal microscopy

## Abstract

Conjunctival goblet cells (GCs) are specialized epithelial cells that secrete mucins onto the ocular surface to maintain the wet environment. Assessment of GCs is important because various ocular surface diseases are associated with their loss. Although there are GC assessment methods available, the current methods are either invasive or difficult to use. In this report, we developed a simple and non-invasive GC assessment method based on fluorescence imaging. Moxifloxacin ophthalmic solution was used to label GCs via topical administration, and then various fluorescence microscopies could image GCs in high contrasts. Fluorescence imaging of GCs in the mouse conjunctiva was confirmed by both confocal reflection microscopy and histology with Periodic acid-Schiff (PAS) labeling. Real-time *in-vivo* conjunctival GC imaging was demonstrated in a rat model by using both confocal fluorescence microscopy and simple wide-field fluorescence microscopy. Different GC densities were observed in the forniceal and bulbar conjunctivas of the rat eye. Moxifloxacin based fluorescence imaging provides high-contrast images of conjunctival GCs non-invasively and could be useful for the study or diagnosis of GC related ocular surface diseases.

## Introduction

Goblet cells (GCs) are specialized epithelial cells that secrete gel-forming mucins. GCs are found intercalated within the epithelia of the respiratory tract, gastrointestinal tract, and conjunctiva^1,2^. Conjunctival GCs are the source of mucins that form the innermost layer of the tear film and are essential for maintaining the wettability on the ocular surface. Mucins reduce friction of the eyeball and protect the eyeball from harmful substances and pathogens^3^. Thus, the decrease of either mucins or GCs was used as an index to measure the severity of various mucin-deficient ocular diseases^4,5^. GC loss is associated with vision-threatening corneal complications such as ocular graft versus host disease (GVHD) and Stevens-Johnson syndrome^6^. Dry eye is a multifactorial disease and GC loss was found during progression. GCs are an important indicator of chemical burn injury and healing after that as well^7–9^. Thus, GC assessment methods may play an important role in the diagnosis of various ocular surface complications. Longitudinal monitoring of GC density changes may be also helpful in post-treatment follow-up.

Impression cytology (IC) is the current standard technique of GC assessment^10,11^. IC involves the application of a cellulose acetate filter onto the ocular surface including the conjunctiva to extract the ocular surface epithelium. The extracted IC samples are stained with Periodic acid-Schiff (PAS) to visualize and analyze GCs and GC density^12^. IC is mildly invasive, and its procedure is not standardized. Frequent sampling using IC can lead to the surface deterioration of the damaged eye. Recently, laser scanning confocal reflection microscopy (CRM) was used as a non-invasive assessment method of GCs to avoid the complications of IC^13^. CRM visualized conjunctival GCs to be large sized cells with relatively high and uniform reflectivity compared to other epithelial cells^13^. However, CRM provides relatively low image contrasts, because the differences in light reflection between goblet cells and other cells are not sufficient especially in damaged eyes. Therefore, CRM examination of GCs requires some training to recognize all the cells on the ocular surface including GCs. A non-invasive and high-contrast imaging method of conjunctival GCs will be useful. We tested fluorescence microscopy with moxifloxacin as a new GC imaging method in this study.

Previously we reported fluorescence tissue imaging with moxifloxacin ophthalmic solution as a cell labeling agent^14^. Moxifloxacin is one of 4^th^ generation fluoroquinolone antibiotics used both to treat and to prevent ocular bacterial infection with good pharmacokinetic properties for tissue penetration, and it has intrinsic fluorescence under ultraviolet (UV) excitation^15^. Its alternative usage as the cell labeling agent was demonstrated with fluorescence microscopy techniques including nonlinear two-photon microscopy (TPM) and confocal fluorescence microscopy (CFM)^14,16^. Moxifloxacin based fluorescence microscopies could visualize cells within tissues in enhanced contrasts. Moxifloxacin usually labels all the cells nonspecifically, but we recently reported that moxifloxacin-based TPM could visualize Paneth cells in the small intestine specifically with strong fluorescence compared to other cells^17^. Paneth cells are granule secreting cells and their granules were labeled strongly by moxifloxacin. Since GCs are secretive cells similarly to Paneth cells, we assumed that GCs could be specifically labeled by moxifloxacin as well. Our preliminary results showed the visualization of GCs in the mouse colon.

In this study, moxifloxacin-based fluorescence imaging was tested as a non-invasive and high-contrast imaging method of conjunctival GCs. Two 3D fluorescence microscopy methods of TPM and CFM were tested in freshly excised *ex-vivo* mouse conjunctiva tissues. CRM was used together with the 3D fluorescence microscopies as the standard GC imaging method. The imaged tissue specimens were processed for histology. After the verification of fluorescence imaging in the *ex-vivo* mouse conjunctivas, *in-vivo* fluorescence imaging of conjunctival GCs was tried in a rat model under gas anesthesia. CFM and wide-field fluorescence microscopy (WFFM) were used for real-time imaging of the rat conjunctiva.

## Results

### Moxifloxacin-based fluorescence imaging of the mouse conjunctiva, *ex-vivo*

**Figure 1:**
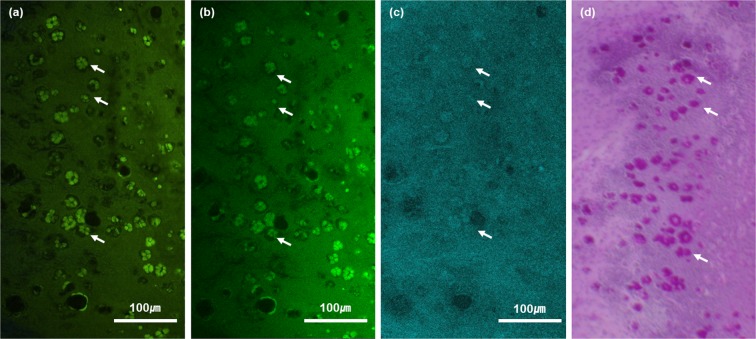
*En-face* TPM, CFM, CRM and histological images of the *ex-vivo* mouse conjunctiva on the surface. (**a**–**c**) moxifloxacin-based TPM and CFM images, and an CRM image of the same mouse conjunctiva tissue respectively. Clusters of highly fluorescent and reflective cells are marked with white arrows (**d**) a PAS histological image of the same conjunctiva tissue. Conjunctival goblet cells were labeled in red color (white-arrow).

Moxifloxacin based fluorescence imaging of conjunctival GCs was tested in *ex-vivo* mouse conjunctiva tissues. The freshly excised mouse conjunctiva tissues were imaged by both TPM and CFM based on fluorescence and then by CRM based on reflectance. After the imaging was completed, the tissues were processed for histology. The results of imaging and histology were presented in Fig. 1. Representative single-plane en-face images on the surface of a mouse conjunctiva tissue were presented. Both TPM and CFM images showed clusters of cells expressing relatively high moxifloxacin fluorescence among epithelial cells expressing relatively weak fluorescence on the conjunctiva surface. The strongly fluorescent cells formed a specific arrangement, similar to a rosette form found in the conjunctival GC clusters of mouse conjunctiva histology^18^. These cell clusters were sparsely distributed. A corresponding CRM image of the mouse conjunctiva showed that the fluorescent cell clusters had relatively high reflectivity compared to the surrounding epithelial cells, indicating conjunctival GCs. Finally, a corresponding en-face PAS histological image showed clusters of conjunctival GCs in similar shapes to the ones in both the fluorescence and reflection images. Spatial distribution of GCs in the histological image was not matched well with the ones in the fluorescence and reflection images. The discrepancy was attributed to mismatch of the sectioning plane with the imaging plane and sample deformation during histology processing.

**Figure 2:**
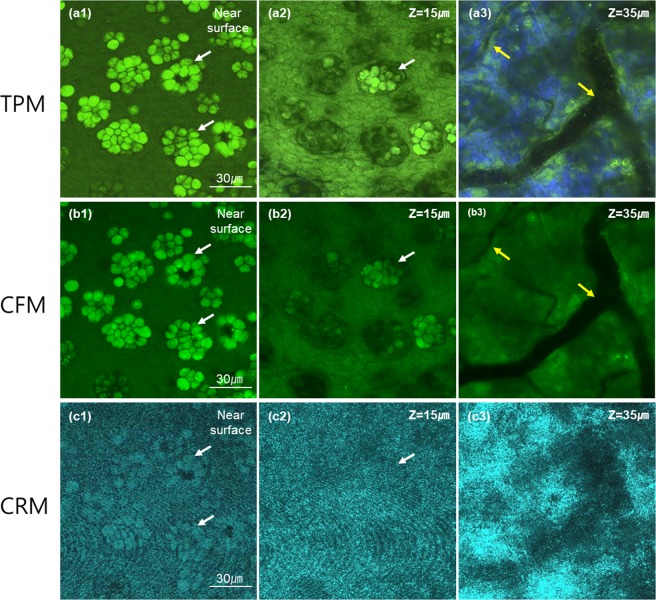
3D TPM, CFM and CRM images of an *ex-vivo* mouse conjunctiva tissue. (**a**–**c**) moxifloxacin-based TPM and CFM images, and an CRM image of the same mouse conjunctiva tissue respectively. Three *en-face* images at different depths of the near surface, 15 μm and 35 μm deep from the surface are presented. The first two superficial images showed the epithelium and the last one image showed the substantia propria. Green color in both TPM and CFM images represented moxifloxacin fluorescence and blue color in the TPM image represented SHG. White and yellow arrows marked goblet cell clusters and blood vessels, respectively.

TPM, CFM, and CRM images in Fig. 1 were single-plane images on the surface from 3D images. The 3D images captured other structures of the mouse conjunctiva below the surface, and their images at several different depths were presented in Fig. 2. 3D TPM images of the mouse conjunctiva showed GCs coming out from the surface (Fig. 2. a1), and then both epithelial cells and GCs at 15 μm deep from the first image plane (Fig. 2. a2). At 35 μm deep from the first image plane, TPM showed the substantia propria below the superficial epithelium (Fig. 2. a3). In the substantia propria, TPM visualized collagen in the extra-cellular matrix (ECM) by second harmonic generation (SHG), blood vessels with no fluorescence expression, and other cells with moxifloxacin fluorescence. 3D CFM images showed cellular structures similar to the TPM images except the SHG contrast. These structures included both protruding GCs with strong fluorescence among weakly fluorescent epithelial cells in the epithelium, and cellular structures in the substantia propria including blood vessels and other cells. 3D CRM images showed highly reflective GCs among less reflective epithelial cells in the epithelium, and highly scattering substantia propria with blood vessels at 3 different depths.

### Moxifloxacin based CFM imaging of the rat conjunctiva, *in vivo*

**Figure 3:**
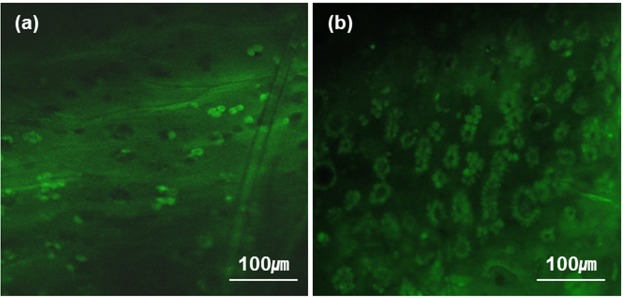
Real-time CFM images of the rat conjunctiva, *in-vivo*. (**a**,**b**) CFM images of the bulbar and forniceal conjunctiva, respectively.

After the fluorescence imaging of conjunctival GCs in the *ex-vivo* mouse conjunctiva, *in-vivo* fluorescence imaging was attempted on the rat conjunctiva under gas anesthesia. CFM was used in the *in-vivo* imaging, because it operates at a relatively high imaging speeds to avoid the motion artifacts such as breathing. Representative CFM images of both the bulbar and forniceal conjunctivas were presented in Fig. 3. Real-time CFM videos of the rat bulbar and forniceal conjunctivas were presented as Supplementary video 1 and 2, respectively. CFM visualized GCs in conjunctival zones. GCs in the rat conjunctiva appeared as clusters in the same way as the ones in the *ex-vivo* mouse conjunctiva. CFM image of the bulbar conjunctiva showed sparse GC distribution, while the forniceal conjunctiva showed denser GC distribution. CFM images of the rat conjunctiva were analyzed to get information of GC density. GC density and GC cluster density in the conjunctiva were obtained after a series of image processing and analysis procedures. GC density and GC cluster density in the forniceal conjunctiva were 2808 ± 250 cells/mm^2^ and 746 ± 103 clusters/mm^2^, respectively. The ones in the bulbar conjunctiva were 385 ± 166 cells/mm^2^ and 311 ± 104 clusters/mm^2^, respectively. The GC cluster densities found in the forniceal and bulbar conjunctivas of the rat eye via the image analysis were consistent with the result of a previous report^18^.

**Figure 4:**
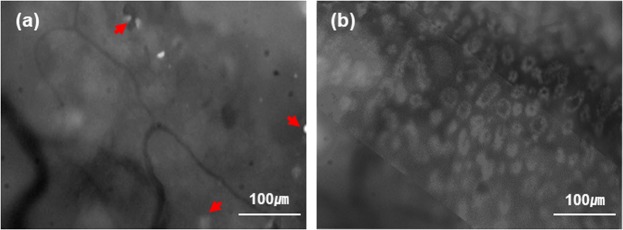
WFFM images of the rat conjunctiva, *in-vivo*. (**a**) a WFFM image of the bulbar conjunctiva, (**b**) a WFFM image of the fornix conjunctiva.

Real-time CFM visualized conjunctival GCs clearly owing to their strong moxifloxacin fluorescence compared to that of surrounding epithelial cells. Because conjunctival GCs were distributed as mono layer on the surface and expressed strong fluorescence compared to other cells, they could be imaged by a simple wide-field fluorescence microscopy (WFFM) which does not have a 3D resolution. WFFM imaging of GCs was attempted on the rat model under gas anesthesia and results were presented in Fig. 4. WFFM images of both the bulbar and forniceal conjunctivas indeed showed GC clusters, although there were relatively high background intensities in the images. The background signals were probably from different depths of the conjunctiva. Although the contrast of WFFM images was low compared to CFM images, GCs were still visible by the simple fluorescence microscopy. WFFM images showed different GC densities in the bulbar and forniceal conjunctivas, as expected. *In-vivo* real-time imaging of GCs in the rat conjunctiva was successfully demonstrated by using both CFM and WFFM.

## Discussions and Conclusions

Fluorescence imaging of conjunctival GCs with moxifloxacin labeling was successfully demonstrated in this study. Moxifloxacin ophthalmic solution labeled GCs stronger than the other epithelial cells in the conjunctiva so that GCs were clearly visualized by fluorescence microscopies. 3D fluorescence imaging of the *ex-vivo* mouse conjunctiva showed specifically arranged clusters of fluorescent cells, which were confirmed to be GCs by the standard CRM imaging and PAS histology. *In-vivo* real-time fluorescence imaging was conducted in the rat conjunctiva. Both CFM and WFFM with moxifloxacin administration could image conjunctival GCs in the rat eye. GCs were distributed densely in the forniceal conjunctiva and relatively sparsely in the bulbar conjunctiva, and the spatial variation of GC density was consistent with literature^18^.

Fluorescence microscopies with moxifloxacin labeling have several advantages over the current non-invasive imaging method, which is CRM. CRM detects GCs based on the relatively high reflectivity and large cell size compared to the surrounding epithelial cells. However, this information is not specific, and the image contrast may not be good enough in case of damaged conjunctiva. Therefore, CRM examination of GCs on the ocular surface requires some experience. On the other hand, fluorescence microscopies provided high-contrast images of conjunctival GCs with moxifloxacin labeling. Quantitative GC density analysis could be easily performed by processing high-contrast GC images. WFFM, which is a simple fluorescence microscopy method without 3D resolution, could visualize GCs on the surface of conjunctiva. WFFM used an air objective lens with 8 mm working distance for conjunctival GC imaging instead of an immersion objective lens which is typically used for CRM. Therefore, Fluorescence microscopy methods could be more convenient in the assessment of conjunctival GCs than CRM.

Moxifloxacin based fluorescence microscopy methods is applicable to human patients in principle by using the FDA-approved moxifloxacin ophthalmic solution for labeling. One concern for using fluorescence microscopy in human patients is photo-toxicity of excitation light. Fluorescence microscopy typically uses short wavelength light in the range of ultraviolet (UV) and short visible light for fluorophore excitation, and 405 nm excitation light was used for GC imaging in the study. Because 405 nm is short visible wavelength (violet), too much exposure could induce photo-toxicity. However, excitation energy of approximately 0.5–3 J/cm^2^ was used for GC imaging in the current study and the energy level was much less than the damage threshold, which was measured to be approximately 50 J/cm^2^ based on cell studies^19^. Therefore, we considered that the fluorescence GC imaging methods are safe and applicable to human as well. We did not notice any damage in both the mouse and rat eyes during and after fluorescence imaging, although more careful safety study would be needed before human trial. Additional safety concern of photo-toxicity by 405 nm excitation light onto other parts of the eye such as the cornea and retina need to be addressed as well. Efforts should be made to minimize the exposure of excitation light during imaging by utilizing safety devices such as shutters and others.

The current imaging protocol of fluorescence microscopies has not been optimized and there are rooms for improvement. The current imaging field of view (FOV) was approximately 1 mm on one side, and FOV could be increased by using low magnification objective lenses. 5x or 10x objective lenses will provide FOV of 4 mm and 2 mm on one side, respectively. The current imaging speeds was 3–5 frames per second (fps) maximum for CFM and 3 fps maximum for WFFM, and the excitation powers were still below the damage threshold. Therefore, the imaging speed can be increased by either increasing the excitation power or by using cameras with the higher sensitivities. Fluorescence microscopies could capture only a small region of the conjunctiva in focus at a time, because the conjunctiva is highly curved. The imaging plane could be easily slanted or mismatched with the conjunctiva surface. In order to capture GCs on the entire conjunctival surface in focus, sequential imaging with a stepwise axial translation of the imaging plane would be needed. Therefore, an auto-focusing mechanism, an axial translation mechanism, and high imaging speeds would be useful for robust imaging and examination of conjunctival GCs and GC density.

CRM has been adapted to daily ophthalmology clinics through long development and commercialization. Confocal imaging on the frontal eye surface can be conveniently done, although it has limitations in the GC assessment. Moxifloxacin based fluorescence imaging methods are new and will need a long period of development and verification in order to be adapted to daily ophthalmology clinics in the future, just like CRM. However, the new fluorescence imaging methods have several advantages over the clinically established CRM in principle in the GCs assessment by providing high-contrast images in simple setting. Also, CFM can be incorporated into the established CRM by adding an additional fluorescence mode to the existing reflection mode. Next steps will be tests of the fluorescence GC imaging methods in animal models of specific diseases associated with conjunctival GC loss for verification and in large animal models such as rabbits.

This study demonstrated high-contrast fluorescence imaging of conjunctival GCs in both *ex-vivo* mouse and *in-vivo* rat models. Moxifloxacin based fluorescence imaging is promising as the conjunctival GC assessment method by providing high-contrast and high-speed images non-invasively.

## Methods

### Subjects and sample labeling

15-week-old SKH1-Hrhr male mice (4 in total) and a 15-month old rat were used for the *ex-vivo* and *in-vivo* imaging experiments, respectively. Moxifloxacin ophthalmic solution (Vigamox, Alcon Laboratories, Fort Worth, USA) was used as a labeling agent. For the *ex-vivo* imaging, mice were euthanized by cervical dislocation during gas anesthesia and then the conjunctiva tissues were excised. The excised mouse conjunctiva tissues were immersed in moxifloxacin ophthalmic solution for 3–5 min, and then were imaged by using both TPM, CFM, and CRM. After the imaging, the mouse conjunctiva specimens were fixed with 4% formaldehyde solution for histology processing. For the *in-*vivo imaging, the rat was gas anesthetized and held by a custom rat eye holder consisting a stereotactic device with ear bars. The left eye was instilled with drops of moxifloxacin ophthalmic solution and incubated for 3–5 minutes. Eyelids were closed during the incubation time to prevent from drying. For the imaging, the eyelid was rolled open with a cotton tip and held with taping. In case of the bulbar conjunctiva, the area between the superior bulbar and the nasal bulbar was imaged. All the animal experiment procedures were approved by the Institutional Animal Care & Use Committee at Pohang University of Science and Technology (IACUC, approval number: POSTECH-2015-0030-R2) and were conducted in accordance with the guidelines.

### Fluorescence imaging

TPM, CFM, and CRM imaging were conducted by using a Leica SP5 confocal and multiphoton microscope (Leica TCS SP5 II) equipped with continuous wave (CW) lasers of various wavelengths and a tunable Ti-Sapphire laser (Chameleon Vision II, Coherent) for confocal microscopy and TPM, respectively. TPM used near-infrared (NIR) wavelengths instead of ultraviolet (UV) via two-photon excitation and could image deeper in turbid tissues than CFM^20^. Three-photon microscopy with moxifloxacin was demonstrated with the higher imaging depth than TPM^21^. Excitation wavelengths of CFM and TPM were 405 nm and 800 nm, respectively for moxifloxacin excitation. Moxifloxacin has single-photon excitation peaks in ultra-violet (UV) wavelengths: first and second peaks at 290 nm and 340 nm, respectively. CFM used 405 nm as the excitation wavelength owing to the availability of laser wavelength, although 405 nm was off from the peak excitation wavelength. CFM could image faster than TPM, because single-photon excitation is much more efficient than two-photon excitation. CRM used 633 nm wavelength for imaging. A 20x objective lens (HCX APO L 20x/1.00 W, 15507701, Leica) was used, and typical images had 775 × 775 μm imaging field of view (FOV) consisting of either 512 × 512 pixels or 1024 × 1024 pixels. Emission light from the specimen was collected in the wavelength range from 430 nm to 650 nm in both CFM and TPM. The imaging speed of the *ex-vivo* conjunctiva specimens was 1.4 frames per second (FPS) in all the microscopies by adjusting excitation or illumination laser powers. Excitation laser power for TPM was approximately 30 mW on the sample surface and increased up to 90 mW with depth, and the one for CFM was approximately 0.1 mW on the sample surface and increased up to 0.9 mW with depth. Illumination power for CRM was 0.1 mW.

For *in-vivo* imaging of the conjunctival GCs, both CFM and WFFM were used. CFM configuration for *in-vivo* imaging was the same as the case for *ex-vivo* imaging, except the excitation power and imaging speed. Relatively high imaging speeds of 3–7 frames/s were used to avoid motion artifacts such as breathing motion, and excitation power for the high-speed imaging was 2.3 mW. WFFM imaging was conducted by using a Leica macroscope (Z16APO A) equipped with a mercury lamp as the excitation light source and a monochromatic camera (DMC6200). 405 nm excitation light was generated by using an excitation filter in the light source. Emission light was collected by using an emission filter transmitting from 430 nm to 650 nm. The imaging FOV was 500 × 500 μm by using a 20x objective lens (HCX APO L 20x/1.00 W, 15507701, Leica), and the imaging speed was 3 frames/s.

### Periodic acid schiff (PAS) histology

The imaged mouse conjunctival tissues were fixed in 4% formaldehyde solution for 24 hours, and then made into paraffin blocks. From the plane where the paraffin block tissue was first exposed, a depth range down to 30 μm~50 μm was sectioned and stained with Periodic Acid Schiff (PAS). The tissue samples were tilted, so the actual depth of the section and the depth from the surface could be different. The above histology procedure was conducted in the pathology department of Asan Medical Center (AMC) in Seoul.

### Analysis of goblet cell (GC) density

**Figure 5:**
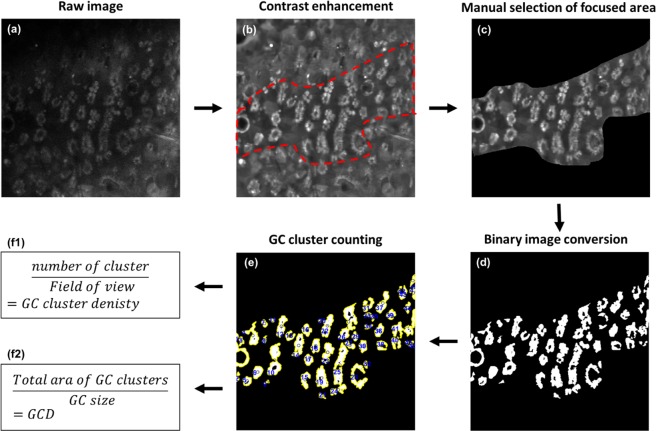
Image processing and analysis flowchart. (**a**) a raw CFM image, (**b**) image enhancement by using BM3D smoothing filter and applying local contrast adjustment, (**c**) manual selection of focused area in the image, (**d**) binary image conversion, (**e**) GC cluster counting, (**f**) GC density and GC cluster density analysis.

GC density was analyzed from the *in-vivo* CFM images of rat conjunctiva. All the image processing and analysis was conducted by using Matlab (Mathoworks, Natick MA), and the flow chart of image processing procedures is depicted in Fig. 5. (1) The GC images were enhanced and smoothened by applying the BM3D image filter^22^. (2) The image contrast was adjusted by using local threshold values obtained from adaptive threshold analysis. (3) In-focus region was manually selected from the GC image, then the intensity image in the in-focus region was converted to a binary image with proper manual thresholding. (4) The number of GCs was calculated by dividing the area of total GC pixels with the average size of GCs, and the density of GCs was calculated by dividing the number of GCs with the in-focus area. (5) The number of GC clusters was analyzed by using a boundary tracing function, and the density of GC clusters was calculated by dividing the number of clusters with the in-focus area. GC size was calculated based on the information of GC diameter, 10.5 μm^23^.

## Supplementary information

Supplememtary information for in vivo fluorescence imaging of conjunctival goblet cells

Real-time CFM video of the rat forniceal conjunctiva, in-vivo

Real-time CFM video of the rat bulbar conjunctiva, in-vivo

## Data Availability

The datasets generated during and/or analyzed during the current study are available from the corresponding author on reasonable request.
