# Supplementary material for: In vivo fluorescence imaging of conjunctival goblet cells
Source: Sci Rep. 2019 Oct 29;9:15457. doi: 10.1038/s41598-019-51893-4 (PMC6820527; doi:10.1038/s41598-019-51893-4)
Supplement: Supplementary file 1 — Supplememtary information for in vivo fluorescence imaging of conjunctival goblet cells [file 41598_2019_51893_MOESM1_ESM.pdf]

Supplementary Information

In vivo fluorescence imaging of conjunctival goblet cells

Seonghan Kim[1], Seunghun Lee[1], Hoonchul Chang[2], Moses Kim[3], Myoung Joon Kim[3,4,*,]
Ki Hean Kim[1,2,*]

Affiliations:
[1]Department of Mechanical Engineering, Pohang University of Science and Technology, 77 Cheongam-ro, Nam-gu, Pohang, Gyeoungbuk 37673, Rep. of Korea

[2]Division of Integrative Biosciences and Biotechnology, Pohang University of Science and Technology, 77 Cheongam-ro, Nam-gu, Pohang, Gyeoungbuk 37673, Rep. of Korea

[3]Department of Ophthalmology, Ulsan College of Medicine, Asan Medical Center, 88 Olympic-ro 43-gil, Songpa-gu, Seoul 05505, Rep. of Korea

[4]SNU Seoul Eye Clinic, 624 Gangnam-daero, Gangnam-gu, Seoul 06035, Rep. of Korea

Email addresses: Seonghan Kim (seonghan@postech.ac.kr), Seunghun Lee (laserman@postech.ac.kr), Hoonchul Chang (hcjang1108@gmail.com), Moses Kim (bswc28@hanmail.net), Myoung Joon Kim (mjmjkim@gmail.com), Ki Hean Kim (kiheankim@postech.ac.kr),

*: corresponding authors

Contents

List of Supplementary Videos

**Supplementary Video 1**	Real-time confocal fluorescence microscopy (CFM) video of the rat bulbar conjunctiva, *in-vivo*
**Supplementary Video 2**	Real-time confocal fluorescence microscopy (CFM) video of the rat forniceal conjunctiva, *in-vivo*

Supplementary Video & Legends

Supplementary Video 1: Real-time confocal fluorescence microscopy (CFM) video of the rat bulbar conjunctiva, in-vivo. (Corresponding to Fig. 3(a))

The image size consists of 512 × 512 pixels covering a field-of-view (FOV) of 387.5 μm × 387.5 μm with a Imaging speed was 7.3 frames/s. Color coding for emission fluorescence was as follows: 430 ~ 650 nm (*green*).

Supplementary Video 2: Real-time confocal fluorescence microscopy (CFM) video of the rat forniceal conjunctiva, in-vivo. (Corresponding to Fig. 3(b))

The image size consists of 512 × 512 pixels covering a field-of-view (FOV) of 387.5 μm × 387.5 μm with a Imaging speed was 7.3 frames/s. Color coding for emission fluorescence was as follows: 430 ~ 650 nm (*green*).
